# Influence of Homogenizing Methodology on Mechanical and Tribological Performance of Powder Metallurgy Processed Titanium Composites Reinforced by Graphene Nanoplatelets

**DOI:** 10.3390/molecules27092666

**Published:** 2022-04-21

**Authors:** Sultan Mahmood, Amjad Iqbal, Rafi-ud-Din, Abdul Wadood, Abdul Mateen, Muhammad Amin, Ibrahim S. Yahia, Heba Y. Zahran

**Affiliations:** 1 Department of Materials Science and Engineering, Institute of Space Technology, Islamabad 44000, Pakistan; ranasmahmood@gmail.com (S.M.); abdul.wadood@mail.ist.edu.pk (A.W.); 2 Department of Materials Technologies, Faculty of Materials Engineering, Silesian University of Technology, Gliwice 44-100, Poland; 3 CEMMPRE—Centre for Mechanical Engineering Materials and Processes, Department of Mechanical Engineering, University of Coimbra, Rua Luı’s Reis Santos, 3030-788 Coimbra, Portugal; 4 Materials Division, Pakistan Institute of Nuclear Science and Technology, Islamabad 45650, Pakistan; rafiuddin@pinstech.org.pk; 5 Department of Materials Science and Engineering, Pak-Austria Fachhochschule Institute of Applied Sciences and Technology, Haripur 22621, Pakistan; abdul.mateen@fcm3.paf-iast.edu.pk; 6 Department of Energy System Engineering Seoul, Seoul National University, Seoul 08826, Korea; amin7818@snu.ac.kr; 7 Laboratory of Nano-Smart Materials for Science and Technology (LNSMST), Department of Physics, Faculty of Science, King Khalid University, P.O. Box 9004, Abha 61413, Saudi Arabia; dr_isyahia@yahoo.com (I.S.Y.); dr_hyzahran@yahoo.com (H.Y.Z.); 8 Research Center for Advanced Materials Science (RCAMS), King Khalid University, P.O. Box 9004, Abha 61413, Saudi Arabia; 9 Nanoscience Laboratory for Environmental and Biomedical Applications (NLEBA), Semiconductor Lab., Metallurgical Lab.1, Department of Physics, Faculty of Education, Ain Shams University, Roxy, Cairo 11757, Egypt

**Keywords:** titanium, graphene nanoplatelets, titanium matrix composites, morphology, mechanical properties, tribological properties

## Abstract

In the present work, 0.25 wt%GNP-Ti composites were prepared through powder metallurgy route by adopting three types of mixing modes to investigate the extent of mixing on the mechanical and tribological properties. Dry ball milling, wet ball milling, and rotator mixing were independently employed to homogenize the composite constituents. Three types of composite powders obtained were subsequently sintered into composite pellets by cold compaction followed by vacuum sintering. Morphological investigation of composite powders performed by SEM revealed better homogenization of GNPs in Ti matrix for dry ball milled composite powder, whereas wet ball milled and rotator mixed composite powders showed aggregation and bundling of GNPs. Micro Vickers hardness of composites produced via dry ball milling is 4.56% and 15.7% higher than wet ball milled and rotator mixed samples, respectively. Wear test performed by pin-on-disk tribometer showed higher wear loss for wet ball milled and rotator mixed composites in comparison to dry ball milled.

## 1. Introduction

Metal matrix composites (MMCs) have been regarded as an essential class of engineering materials due to their better thermal and mechanical performance [1,2]. Conventionally, titanium and its alloys seem to be a better choice for automotive, aerospace, and bio-medical applications owing to the medium density, moderate mechanical strength, good corrosion and oxidation resistance, better fracture toughness, and biocompatibility. However, their use in many applications is limited because of poor wear resistance and lower electrical and thermal properties [3,4]. Poor tribological performance of titanium, for example, inferior fretting behavior and lower wear resistance, are chiefly due to its low thermal conductivity and less plastic shearing resistance [5]. In addition, many structural and engineering applications require a higher degree of mechanical strength. To overcome such deficiencies in titanium, numerous reinforcement materials have been incorporated to develop titanium matrix composites (TMCs) [6]. Various ceramic reinforcements have been reported in the literature to improve the strength and wear resistance of Ti and its alloys. Shufeng et al. investigated the synergic effect of in situ synthesized TiC-TiB reinforcements on microstructure and mechanical properties of TMCs and observed a 1.7 times increase in tensile strength accompanying a 12.5% decline in ductility for 13.6 vol% reinforcement [7]. Ti/TiC nanocomposites were synthesized by Gu et al., who found optimum content of TiC as 12.5 wt% to achieve a 2 times increase in microhardness and considerably low coefficient of friction (COF) and wear rate [8]. Wang et al. achieved 3.5 times higher compressive strength at the expense of a 20% decline in strain to failure for in situ synthesized nanometric TiC-Ti composites compared to un-reinforced Ti [9]. Al_2_O_3_-Ti nanocomposites were produced by Zarghani et al. through friction stir processing and noticed 1.5 times enhancement in compressive yield strength for 3.9 vol% Al_2_O_3_-Ti in comparison to as received Ti [10]. Huang et al. obtained 1.36 times increase in tensile strength for 5 vol%TiB nanowires reinforced Ti-6Al-4V composites compared to that of un-reinforced alloy [11]. Pan et al. developed in situ formed TiC-Ti composites and showed 62% higher nano-indentation hardness and 30% lower COF than pure Ti [12]. Recently, Jin et al. found a 68.6% increase in hardness and 32.6% improvement in wear resistance of TiB2-Ti composites produced by selective laser melting [13]. However, reported enhancement in mechanical strength and wear resistance is achieved at the cost of lightweight, toughness, thermal, and electrical properties because ceramics have higher densities, low fracture toughness, and poor thermal and electrical conductivity than titanium [14]. 

In view of the above, lightweight reinforcement materials having high strength, toughness, and electrical and thermal conductivity are indispensable in realizing the goal of producing titanium composites for aerospace, automotive, and bio-medical applications. In this perspective, carbonaceous materials, for example, carbon fiber (CF), nano-diamond (ND), carbon nanotubes (CNTs), and graphene nanoplatelets (GNPs), emerged as an ideal choice thanks to their superior chemical and physical characteristics [15,16]. Nano-diamonds reinforcement (0.35 wt%) in the Ti matrix resulted in a 71.5% reduction in wear rate, as investigated by Saba et al. [17]. CNTs due to the high aspect ratio, high strength, and self-lubrication properties appear to be a promising reinforcement for TMCs [18]. Research conducted by Kuzumaki et al. demonstrated a 5.5 times improvement in hardness of Ti through 0.8 vol% CNTs reinforcement [19]. Kondoh et al. achieved a 1.2 and 1.5 times increase in tensile and yield strength, respectively, for 0.35 wt% CNT-Ti composites without much reduction in the ductility [20]. Moreover, 3 wt% CNT-Ti composites were produced by Xue et al., wherein they confirmed good compressive strengthat elevated temperature when subjected to higher strain [21]. Wang et al. reported 61% compressive strength enhancement for 0.4 wt% CNT-Ti composites [22]. Munir et al. researched on the powder metallurgy processing of CNT-Ti composite and investigated the influence of processing parameters and CNTs content on microstructural, mechanical, and wear properties. Increase in compressive strength by 1.5 times and wear reduction by a factor of 3.4 were reported for 0.5 wt% CNT-Ti composites [23,24,25]. However, among carbonaceous reinforcement materials, graphene has led the race, because it is gifted with low density (1.8 g/cm^3^), high strength (125 GPa), high elastic modulus (1.1 TPa), very good electrical conductivity (2 × 10^8^/ohm/m), remarkable thermal conductivity (5 × 10^3^ W/m/K), very high specific surface (2630 m^2^/g), and outstanding self-lubrication behavior [26]. Graphene is basically a two-dimensional single atomic layer of SP^2^ hybridized carbon atoms arranged in a honeycomb lattice with a bond length of 0.142 nm. Its extraordinary self-lubrication is due to easy sliding on the densely packed surface [27]. However, the synthesis of perfect single layer graphene with ideal properties is not practicable [28]. Therefore, multilayer graphene with more than 10 layers, termed as graphene nanoplatelets (GNPs), retaining most of the single layer properties has been proved to be a more suitable reinforcement choice for composites applications [29].

Therefore keeping in mind the aim to develop lightweight, tough, and wear resistant composites, a systematic literature survey regarding GNPs-Ti composites has been carried out which shows that research in this area is in the initial stage. Prior works synthesized the GNPs-Ti composites through the powder metallurgy (PM) route and explored some significant aspects. Yang et al. produced graphene platelets (GPs) reinforced titanium composite via ball milling, cold compaction, and microwave sintering. They reported an 8% increase in thermal conductivity for 0.4 wt% GP, while the maximum compressive strength was recorded at 0.3 wt% GPs [30]. Song et al. synthesized TMCs reinforced by multilayer graphene (MLGs) through solution ball milling and spark plasma sintering (SPS) and found an optimum content of 0.5 wt% MLGs for improvement in microscopic mechanical properties and scratch resistance of TMCs, but properties declined at 1.5 wt% MLGs. [31]. Cao et al. achieved a 12%, 20%, and 15% increase in tensile strength, yield strength, and elastic modulus, respectively, for 0.5 wt% graphene nanoflakes (GNFs) reinforced Ti-6Al-4V composites fabricated via blending, wet mixing, hot isostatic pressing (HIP), and isothermal forging. Notably, enhancement in mechanical properties was registered without the loss of ductility [32]. Gurbuz et al. developed 0.15, 0.30, 0.45, and 0.60 wt% GNPs-Ti composites by blending, ball milling, cold compaction, and vacuum sintering. Optimized sintering conditions were noticed at 1100 °C, 120 min for 0.15 wt% GNPs-Ti composites, displaying a 86% increase in hardness [33]. Mu et al. have explored the influence of GNPs contents and its dispersion on the mechanical performance of TMCs, in addition to the role of secondary processing such as hot rolling on mechanical properties of GNP-Ti composite, produced through wet ball milling and spark plasma sintering. Tensile strength and yield strength were enhanced to 24% and 27%, respectively, for 0.4 wt% GNPs, whereas for 0.8 wt% GNPs-Ti composite system, the nanoindentation test showed a 96% hardness increase in the direction perpendicular to hot rolling [34,35]. Guo et al. used ball milling and SPS to fabricate Ti-6Al-4V matrix composites reinforced by Ni-P coated GNFs with varying content from 0.25 to 1.5 wt%. They observed a uniform distribution of GNFs and reported a 1.6 times increase in compressive yield stress for 0.5 wt% GNFs while maintaining 34.2% ductility [36]. 

Despite some very encouraging outcomes, the field is still open to explore the full perspective of graphene reinforced titanium matrix composites for their effective use in aerospace, automotive, and bio-medical sectors. Processing of these composites poses some big challenges. Since the first and crucial step in PM is the homogenous mixing of composite constituents to attain optimum property attributes in sintered composite [37], the primary target in developing GNPs-Ti composites is to overcome the challenge of GNPs aggregation in the titanium matrix because GNPs dispersion in metal matrices is even more difficult than CNTs, due to the very high specific surface area of graphene [38]. Therefore, ample homogenization of GNPs-Ti composites powders before consolidation is the most vital process phase. Earlier works focused on PM processing by employing one type of homogenizing approach. Keeping in view the applications demand of TMCs such as landing gear and turbine engine parts for aircraft and bone and dental implants [39,40], there is a need to explore and compare various homogenizing techniques and optimize the best suitable method to reach the targeted attributes, for example, enhanced mechanical and tribological characteristics. To the authors’ best knowledge, no comprehensive study on the investigation of homogenizing methodology’s influence on the mechanical and tribological performance of GNPs-Ti composites has been published so far. 

The main purpose of this research is to produce GNPs-Ti composites with a higher level of mechanical and tribological performance suitable for the longer and proficient functioning of aerospace and bio-medical components. Therefore, this work is primarily designed to develop 0.25 wt% GNP-Ti composites through PM processing by adopting three different homogenizing approaches: dry ball milling, wet ball milling, and rotator mixing. The influence of homogenizing methodologies on morphological variations of composite powders and its connection with the mechanical strength and tribological properties of developed composites have been investigated. 

## 2. Experimental Part

### 2.1. Materials

**Figure 1:**
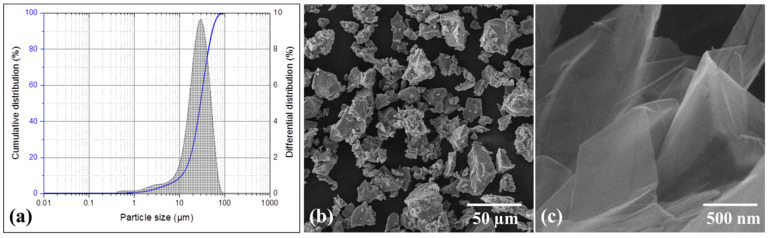
Stating materials: (**a**) particle size analysis of Ti powder; SEM micrographs showing morphology of (**b**) Ti powder and (**c**) GNPs.

Matrix material used in this research is commercially pure titanium powder, having a mean particle size of 28.8 µm and bulk shape morphology as evident in Figure 1a,b. The chemical composition of as received Ti powder is presented in Table 1. For reinforcement, GNPs having an average thickness of 5–25 nm and a length of 1–20 µm are used. The morphology of GNPs is shown in Figure 1c. Both raw materials were purchased from Guangzhou Jiechuang, Co., Ltd. (Guangzhou, Guangdong, China). 

### 2.2. Processing

The solid-state powder metallurgy route is adopted to produce 0.25 wt% GNP-Ti composite samples. In the first step, GNPs are homogenized in Ti powder through three different mixing techniques to obtain three types of composite powders. In the second stage, composite powders are cold pressed followed by vacuum sintering to obtain three types of sample batches. Samples preparation through this route is described in subsequent sections, and processing parameters are summarized in Table 2.

#### 2.2.1. Dry Ball Milling

In this technique, 0.25 wt% GNP-Ti composite powder is obtained through ball milling in dry form by employing a planetary ball mill (PM 4, Retsch, Germany). Besides composite constituents, 0.3 wt% stearic acid (SA) is added as a process control agent (PCA). The function of PCA during mechanical alloying is to avoid cold welding and accelerate the fracturing of powder particles [41]. GNPs and Ti powders in weighed quantity are put into a steel vial (500 mL, ID = 100 mm) together with the tungsten carbide balls as a milling media. Balls of dissimilar sizes (5 mm and 10 mm diameter) are used to bring in more collisions and promote fracturing over cold welding [42]. Ball to powder ratio (BPR) is set at 6:1. Ball milling is performed at 225 rpm for 3 h in dry argon atmosphere to prevent oxidation of freshly produced surfaces [43].

#### 2.2.2. Wet Ball Milling

In wet ball milling, initially, GNPs slurry is prepared by dispersing GNPs in methanol at a ratio of 1 mg/1 mL, via 30 min ultrasonication in a water bath at 60 °C. Secondly, Ti powder slurry is produced by mixing it in methanol at the proportion of 1 g/2 mL by ultrasonication for 30 min in the water bath at 60 °C. In the third step, two slurries are mixed thoroughly by a 30 min magnetic stirring to obtain the composite slurry. Then, composite slurry is charged into a steel vial (500 mL, ID = 100 mm) along with milling media of tungsten carbide balls of 5 mm and 10 mm in diameter. Ball to powder ratio (BPR) is 6:1 and wet ball milling is conducted at 225 rpm for 3 h. Finally, ball milled slurry is dried completely at 100 °C in the oven.

#### 2.2.3. Rotator Mixing

To prepare 0.25 wt% GNP-Ti GNPs composite powder via rotator mixing, first GNPs and Ti powder slurries are prepared as in Section 2.2.2. Then, a rotator mixer with the stainless-steel blade is employed to mix these two slurries at 300 rpm for 3 h. After that, composite slurry prepared is fully dried in the oven at 100 °C.

#### 2.2.4. Consolidation

GNP-Ti composite powders (0.25 wt%) prepared via three mixing methods are consolidated by uniaxial cold compaction and subsequent vacuum sintering. In the first step, composite powders are charged in a steel die with an inner cavity diameter of 19 mm and cold pressed at 400 MPa using a hydraulic press (15-Ton, STENHɸJ). Zinc stearate is incorporated to act as a die lubricant [44]. Successively, pressed compacts termed as green pellets are sintered in a vacuum furnace (Energyen, Korea) at 1100 °C for 2 h at a heating and cooling rate of 10 °C/min under 10^−3^ Pa vacuum.

### 2.3. Characterization

Particle size analysis of as received Ti powder is performed by the laser diffraction particle size analyzer (Mastersizer 3000E, Malvern Instruments, Malvern, UK). The morphology of Ti powder and GNPs is examined by field emission scanning electron microscope (FE-SEM MIRA-III, TESCON, Kohoutovice, Czech Republic) in the secondary electron imaging mode at an accelerating voltage of 20 KV. The purity of Ti powder is analyzed by inductively coupled plasma-optical emission spectroscopy (ICP-OES). This technique is preferred because its detection limits are in the parts per billion (ppb) to parts per million (ppm) range and can analyze trace impurities in metal and alloys. Further, chemical and ionization interferences relatively do not occur in ICP-OES, which gives highly accurate results. Principles of operation, samples detail, and analyzing procedures can be found elsewhere [45,46]. 

Morphological modifications induced in 0.25 wt% GNP-Ti composite powders by homogenizing methods are investigated by FE-SEM in secondary electron imaging mode at an accelerating voltage of 20 KV. Raman spectroscopy (Horiba HR 800 UV) is used to investigate the quality of as received graphene and its structural integrity in composites. A HE-NE laser of 633 nm wavelength is used at a numerical aperture of 50× lens with 600 g/mm grating. Raman scattering is performed at six different points of each sample with a scattering range of 200–3500 cm^−1^. The crystal structure of raw materials and phase composition of composites are analyzed by X-ray diffraction (XRD) technique, using Rigaku X-ray diffractometer with Cu Kα radiation over 2θ scanning range of 20–80°. 

The green density of cold compacted pellets is calculated by the geometrical method, while the sintered density of composite pellets is determined by the Archimedes principle using a densimeter (AU-900S, Dong Guan Hong Tuo Instrument Company, Guangdong, China) having an accuracy of 10^−3^ g. Samples for hardness measurement are prepared by a series of grinding and polishing steps to attain a 1 µm finish. Polished specimens are ultrasonically cleaned in ethanol for 30 min and dried in an oven. Hardness measurement is performed by a Vickers micro-hardness tester (Karl Frank, Wurzburg, Germany) equipped with a pyramid diamond indenter at a load of 980.7 mN for 15 s. As 100% dense composite material fabrication is not possible through the described PM route. Therefore, hardness reading may vary to some extent at different locations of the same sample due to the presence of porosity [47,48]. Because of this, 6 readings were recorded at various points of the sample, and an average was taken. 

Tribological properties of composites are studied by performing the wear test on pin-on-disk tribometer (MT-Spain). Specimens in round shape (18 mm diameter, 6 mm thickness) are prepared through grinding up to 2000 grit finish. Before testing, each specimen is ultrasonically cleaned in ethanol for 30 min, dried in the oven, and weighed carefully using an electronic weighing balance having accuracy up to 4 decimal places. The specimen is fixed at a rotating disk while diamond pin is employed as the counter body. Wear tests are run at 10 N load, 3 mm track radius, 100 rpm, 190 m sliding distance, in the dry state at 25 °C and 55%RH. As a result of the wear test, wear tracks are produced at the sample surface, and the removed material or wear debris is collected. The sample is cleaned, dried, and weighed again to record the wear mass loss. The wear rate is determined as per ASTM G-99. The coefficient of friction (COF) is plotted from machine data up to the sliding distance at which the constant COF value is reached. The wear behavior or wear mechanisms involved are studied by examining the worn surfaces pattern and wear debris morphology through FE-SEM in secondary electron imaging mode at an accelerating voltage of 20 KV. Energy dispersive x-ray analysis (EDX) of worn surfaces and collected wear debris has been performed by FE-SEM.

## 3. Results and Discussion

### 3.1. Morphological Evolution of Composite Powders

**Figure 2:**
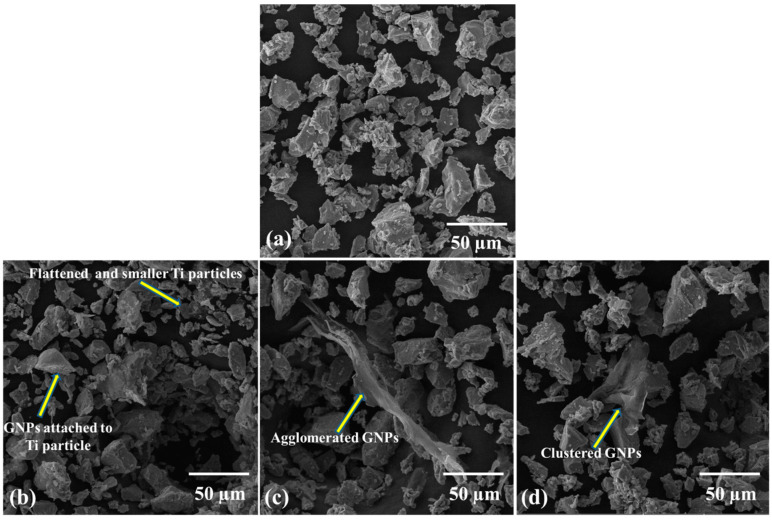
SEM micrographs at magnification of 1 kx showing morphology of (**a**) as received Ti powder, and 0.25 wt% GNP-Ti composite powders mixed through (**b**) dry ball milling, (**c**) wet ball milling, and (**d**) rotator mixing.

**Figure 3:**
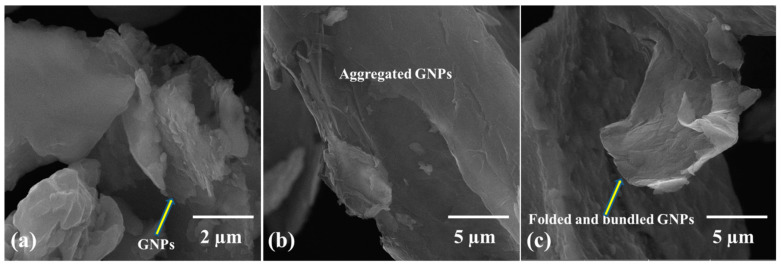
Higher magnification SEM micrographs showing morphology of 0.25 wt% GNP-Ti composite powders mixed through (**a**) dry ball milling (at magnification of 25 kx), (**b**) wet ball milling (at magnification of 10 kx), and (**c**) rotator mixing (at magnification of 10 kx).

Figure 2 shows the morphology of as received Ti and 0.25 wt% GNPs-Ti composite powders prepared via three different mixing techniques. A significant change in the morphology of composite powders has been observed compared to as received Ti powder. In addition, SEM images manifested the dependence of composite powder morphology and GNPs dispersion pattern on the mixing method. In contrast to bulk shaped morphology of unreinforced Ti powder (Figure 2a), dry ball milled composite powder exhibits more flattened and smaller Ti particles (Figure 2b). On the other hand, relatively less reduction in particle size and almost insignificant morphology shift from bulky to flattened has been noticed for both wet ball milled and rotator mixed composite powders (Figure 2c,d). Moreover, the homogenous distribution of GNPs in Ti matrix is also of great importance in connection with final composite properties. In this perspective, different GNPs dispersion patterns were seen concerning the mixing route as obvious in Figure 2 and Figure 3. It can be noticed in Figure 2c and Figure 3b that wet ball milled composite powder present some kind of GNPs agglomeration which appears to be bundled and clustered. Nevertheless, for rotator mixed composite powder, the extent of agglomeration is relatively less, and GNPs seem to be folded as obvious in Figure 2d and Figure 3c. These detected variations in particles morphology and GNPs distribution pattern may be attributed to the different mechanisms and process dynamics of the homogenizing approach. It is expected that during the dry ball milling of composite powder, at certain milling energy and time, a dynamic balance of compression, shear, and impact forces is reached [49]. Therefore, as a result, Ti particles are flattened due to the pressing action of compressive forces, while particles shearing during ball milling yields cracks on the surface, and ultimately, impact forces break down the particles into smaller fragments [50]. In addition, high impact forces during dry ball milling contribute to the attachment of GNPs onto the surface of Ti particles, resulting in better homogenization of the composite powder [35]. Conversely, wet ball milling has a minor impact on the beneficial modification of Ti particle size and shape, in addition to GNPs clustering in Ti matrix. This may be ascribed to the inadequate compressive and impact forces hindered by the wet medium leading to poorer composite powder characteristics [51], whereas for rotator mixed composite powder, particles fragmentation into smaller ones does not happen owing to the nonexistence of grinding media. However, prevailing rotational forces lead to less accumulation of GNPs in Ti matrix in contrast with wet ball milling [52].

### 3.2. Raman Spectroscopy

**Figure 4:**
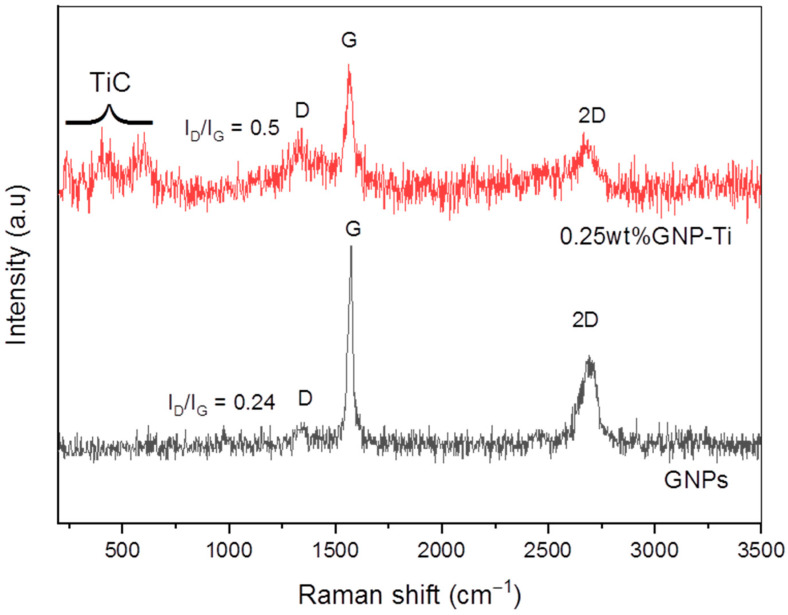
Raman spectra of starting graphene material and 0.25 wt% GNP-Ti composite powder produced via dry ball milling technique.

Effective use of graphene as reinforcement in Ti composites is largely associated with the retention of structural properties during the composites’ synthesis process [53]. Raman spectroscopy is a powerful non-destructive technique to investigate the quality and structural integrity of carbonaceous materials. It gives a characteristic spectrum of any material depending on the vibrational modes of its molecules [54]. Figure 4 represents the Raman spectra of starting graphene and 0.25 wt% GNP-Ti composite powder produced by the dry ball milling method. Spectra reveal typical D, G, and 2D bands of graphene at 1340, 1575, and 2690 cm^−1^, respectively. D-band in spectra is related to the concentration and measure of disorders in C-C bond in graphitic materials. G-band represents the in-plane vibration of C-C bonds and the extent of metallicity or graphitization. 2D-band is associated with multilayer graphene structure [55]. Composite powders also show D, G, and 2D bands peaks at 1340, 1575, and 2690 cm^−1^, respectively, corresponding to starting GNPs peaks. This confirms the presence and survival of un-reacted GNPs in composite powders after homogenizing [23]. Ti peaks are not revealed in the observed spectral range which may be due to the reason that no active vibrational modes are present in Ti to be detected by Raman spectroscopy [56]. Relatively less intensity of the 2D-band than the G-band is observed in spectra of graphene and composite powder as well, which indicates the existence of graphene in multilayers [57]. 

Raman peak intensity ratio (I_D_/I_G_) is the measure of structural defects in graphitic materials [58]. I_D_/I_G_ ratio of starting graphene material is determined as 0.24, whereas for composite powder it is 0.5. This points out the presence of limited defects in as received GNPs. Intensity ratio I_D_/I_G_ is relatively higher for composite powder, which is an obvious indication of induced structural defects in GNPs present in composite powder due to the homogenization process [53]. Further analysis of Raman spectra of 0.25 wt% GNP-Ti composite powders shows peaks at 260, 418, and 605 cm^−1^. These peaks indicate the formation of interfacial compound TiC during milling. This observation is further supported by a relatively higher spectrum background in composite powder than as received GNPs, which is also ascribed to the TiC formation due to harsh ball milling conditions. Similar findings have been reported in earlier works [23,57]. 

### 3.3. XRD Analysis

**Figure 5:**
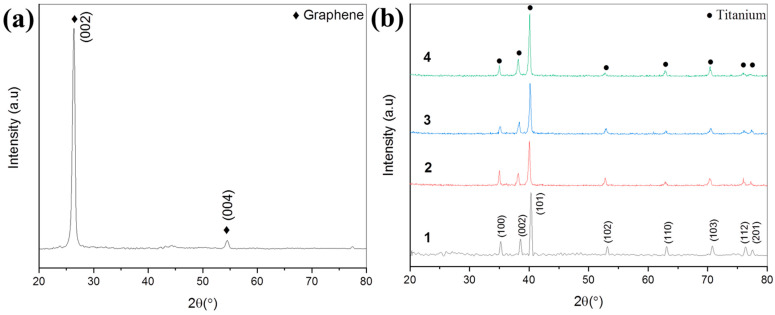
XRD patterns of (**a**) as received GNPs, (**b**) as received Ti (1), 0.25 wt% GNP-Ti composites processed through: dry ball milling (2), wet ball milling (3), and rotator mixing (4).

XRD patterns of as received graphene nanoplatelets, pure titanium, and 0.25 wt% GNP-Ti composites are presented in Figure 5. XRD peaks shown in Figure 5a are characteristic of graphene confirming its quality [59,60], whereas pure Ti and 0.25 wt% GNP-Ti composites exhibited elemental Ti peaks having hexagonal close packed (hcp) structure, as evident in Figure 5b. Ti Peaks are indexed as (001), (002), (101), (102), (110), (103), (112), and (201), which correspond to hexagonal Ti [61]. However, in all composites, peaks corresponding to graphene are not detected because in this study the weight fraction of GNPs is very low (0.25%), whereas XRD has a detection limit for the identification of low concentrations of nano-sized second phase [62]. Further, XRD patterns do not show the peaks of interfacial compound TiC. However, there is a possibility for the formation of nanocrystalline TiC due to the reaction between Ti and graphene during the homogenizing and sintering process [23]. This argument is well supported by the Raman spectroscopy results presented in Section 3.2. However, the identification of very small amounts of TiC in GNP-Ti composites through the XRD technique may not be possible due to the XRD detection constraint of XRD [63].

### 3.4. Density and Sinterability of Composites

As discussed in Section 3.1, the morphology of composite powder and pattern of GNPs dispersion in the matrix are mainly dependent on the mixing method, and consequently, density and sinterability of the composites are also modified. Theoretical densities of the composites were computed by the rule of mixture [64]

(1) $$\rho_{c}=\rho_{GNP}W_{GNP}+\rho_{M}W_{M}$$

where *ρ* is the density; W is the weight fraction, and the subscripts “*C*”, “*GNP*”, and “*M*” denote the composite, GNPs, and Ti matrix, respectively. Theoretical density calculated from the above equation and experimentally measured green and sintered densities are put into the below equation to compute the sinterability (Ø) of composites.

Ø = ρ_s_ − ρ_g_/ρ_th_ − ρ_g_ (2)

where ρ_s__,_ ρ_g_ and ρ_th_ are the sintered, green, and theoretical densities, respectively. Results are shown in Table 3. This suggests that the composite samples processed through dry ball milling display the highest green and sintered density, whereas those processed through wet ball milling display the lowest. Sinterability of composite powder compact depends upon particle size, shape, particle size distribution, and homogenous mixing of composite constituents in addition to sintering conditions [47]. Therefore, dependency of sinterability upon the mixing approach may be linked to the morphology evolution of Ti particles and GNPs dispersion style in the matrix. It is quite evident from Figure 2 that composite powder mixed via dry ball milling presented flattened morphology and a reasonable fraction of smaller particles as well. Moreover, GNPs adherence to Ti particles (Figure 3a) leads to its homogenous dispersion. These aspects have a substantial role in promoting the sintering kinetics through easy and quick material transport [44,65]. Consequently, the sinterability of composites produced via dry ball milling is higher, and the density also exhibits an analogous trend. In the case of wet ball milled and rotator mixed composites, GNPs agglomeration has a negative impact on density. During mixing and consolidation, GNPs aggregation promotes friction between GNPs interlayers and also between GNPs layers and Ti particles. As a consequence, constraint in GNPs rearrangement and reduction in the contact area between GNPs and Ti particles result in relatively higher porosity and lower density [66]. Table 4 presents the density and porosity of various GNP-Ti composites processed through the pressureless sintering method.

### 3.5. Mechanical Strength of Composites

**Figure 6:**
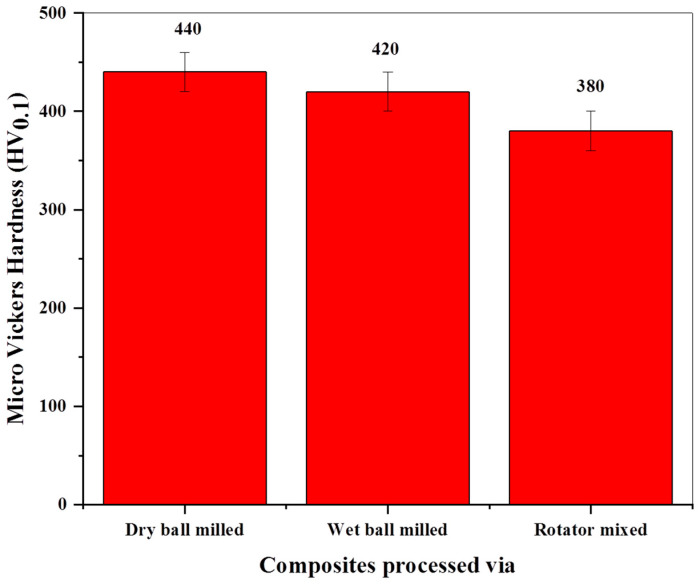
Micro Vickers hardness of 0.25 wt% GNP-Ti composites in relation to mixing method.

The mechanical strength of a material is directly related to its hardness, as reported by many researchers [67,68,69]. Therefore, in this study, hardness measurement is performed on synthesized composites to evaluate their mechanical performance. Figure 6 depicts the hardness of three types of composites concerning homogenizing method. Composites synthesized via dry ball milling exhibit higher hardness than others. This trend points out the dependence of mechanical strength upon the composite constituents homogenizing approach, and this is believed to be caused by two factors, namely, porosity level in sintered composite and GNPs role as nano-reinforcement. It is well known that the strength of a material can be increased by reducing the extent of plastic deformation by restricting the dislocation movement [70,71]. The presence of porosity in the composite compact has a negative effect on its strengthening efficiency. Because pores facilitate easy dislocation movement, a consequently higher degree of plastic deformation results in reduced hardness [72,73], as is the case of composites processed through wet ball milling and rotator mixing in the present work (Figure 6). 

Secondly, the role of nano-metric reinforcements in improving the mechanical strength of composite materials through the well-known strengthening mechanisms is widely accepted [74,75]. Graphene nanoplatelets being the only few layers of atomically thin graphene have proved to be the most effective nano-reinforcement for boosting the strengthening efficiency of metal matrices through the contribution of their strengthening mechanisms [29,76]. These mechanisms are: (i) Hall–Petch strengthening due to the restriction of grain growth by the GNPs pinning effect [77], (ii) Orowan strengthening because of dislocations looping around possibly formed in situ TiC dispersoids [78], (iii) Taylor relationship or enhanced dislocation density (EDD) effect due to the mismatch of elastic modulus (EM) and coefficient of thermal expansion (CTE) between GNPs and Ti matrix [10], (iv) efficient load transfer effect from Ti matrix to GNPs by interfacial shear stress because of high elastic modulus of GNPs and strong interface bond by TiC between GNPs and Ti matrix [74], (v) contribution from dispersion strengthening effect owing to homogenously dispersed GNPs and in situ formed TiC particles in the matrix, and (vi) solid solution strengthening by the interstitial carbon atoms in titanium lattice [79]. However, the strengthening mechanisms’ contribution is largely dependent on GNPs content and their homogenous dispersion in matrix material [80]. Generally, higher GNPs contents favor more strengthening efficiency. However, it has been reported that strength declines beyond 0.5 wt% GNPs due to their agglomeration and clustering [31]. 

It is worth mentioning here that the results obtained in this study are in close agreement with the above argument and prior works, as evident in Table 5. Correlating the hardness results with the morphological evolution study of composite powders, it is obvious that good adherence of GNPs onto the Ti particle surface favors uniform dispersion in the dry ball milled sample (Figure 2b), so the sintered composite displays higher hardness. In contrast, apparent GNPs aggregation and bundling have been observed in wet ball milled and rotator mixed composite powders, respectively (Figure 2c,d), and hence they exhibit relatively less hardness. In view of above, the dry ball milling approach has shown good potential to adequately homogenize the 0.25 wt% GNP-Ti composite constituents and achieve good mechanical strength in the final composite.

### 3.6. Tribological Performance

**Figure 7:**
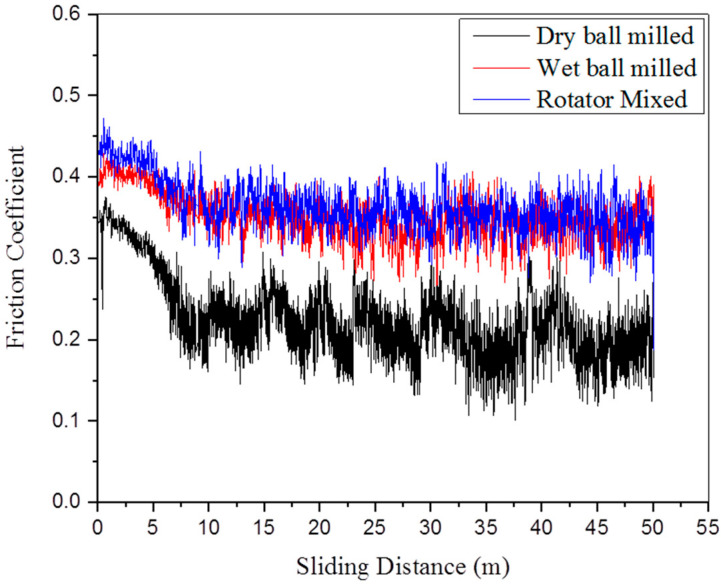
Coefficient of friction plot obtained from wear test of 0.25 wt% GNP-Ti composites in connection with mixing method.

**Figure 8:**
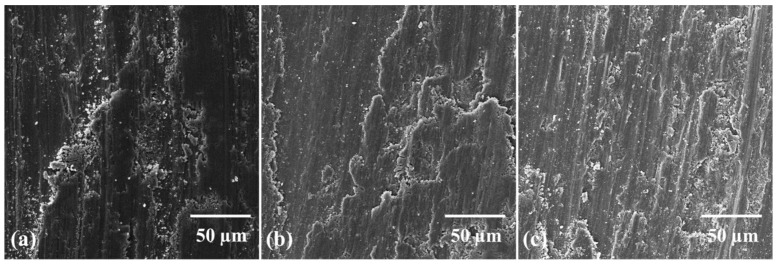
SEM images (at magnification of 1 kx) of worn surface as a result of wear test performed on 0.25 wt% GNP-Ti composites produced via (**a**) dry ball milling, (**b**) wet ball milling, (**c**) rotator mixing.

**Figure 9:**
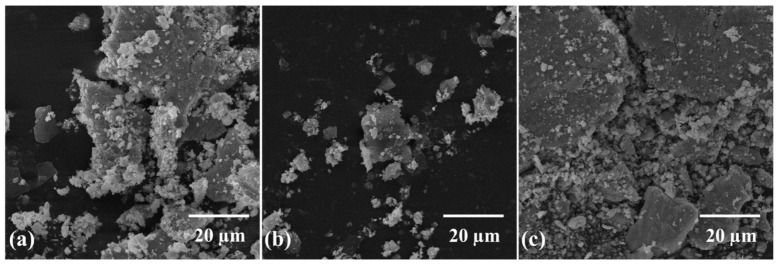
SEM images (at magnification of 3 kx) of wear debris collected after wear test performed on 0.25 wt% GNP-Ti composites prepared through (**a**) dry ball milling, (**b**) wet ball milling, (**c**) rotator mixing.

**Figure 10:**
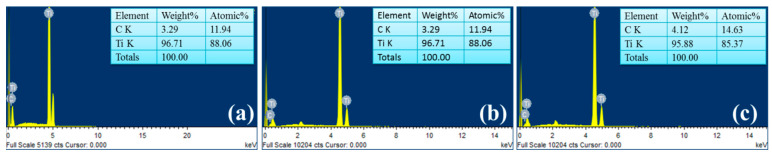
EDX results performed on wear tracks of 0.25 wt% GNP-Ti composites prepared through (**a**) dry ball milling, (**b**) wet ball milling, (**c**) rotator mixing.

**Figure 11:**
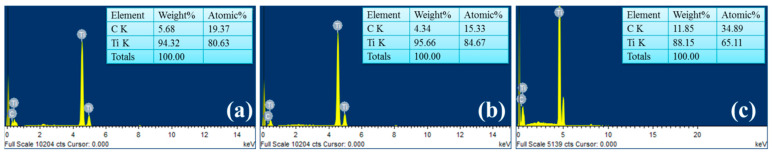
EDX results performed on wear debris of 0.25 wt% GNP-Ti composites prepared through (**a**) dry ball milling, (**b**) wet ball milling, (**c**) rotator mixing.

Table 6 presents the results of tribological testing performed on composite samples, in relation to coefficient of friction (COF) and wear rate. It is inferred from the results that composites processed via dry ball milling present relatively less wear loss than wet ball milled and rotator mixed ones, so they exhibit reduced wear rate and coefficient of friction. As discussed in Section 3.5, the strength of composites is directly related to the degree of sinterability and homogenous GNPs dispersion in the matrix. The hardness of a material is linked to its wear properties in some way [81]. Consequently, for composites produced via dry balling, the extent of plastic deformation will be lower, thereby producing less abrasion, which results in reduced wear loss and consequently lower COF value as evident in Figure 7. On the other hand, a somewhat higher porosity level and GNPs clustering in the Ti matrix promote plastic deformation, thus producing more wear debris, so higher wear loss and COF are recorded for composites processed via wet ball milled and rotator mixing. However, slightly better wear resistance has been observed for rotator mixed composites than wet ball milled composites because of relatively less GNPs agglomeration. Figure 8 shows SEM images of worn surfaces for three types of composite samples. The less plowing effect is observed for the composite produced via the dry ball milling method in comparison with the other two. Delamination of layers occurs because of the adhesive wear mechanism, whereas it is obvious that due to the higher plowing effect and abrasion, the abrasive wear mechanism is operative for the wet ball milled and rotator mixed composite samples. Observations are also well supported by the morphological investigation of wear debris as evident in Figure 9. EDX results presented in Figure 10 and Figure 11 confirmed the absence of wear debris from the counter surface.

It has been found in earlier works that improved tribological performance of GNP reinforced metal and ceramics matrix composites occurs because of the low shear, protective nature, and self-lubrication behavior of graphene [82,83]. Therefore, its homogenous dispersion in metal matrices may result in the formation of a stable and thin dry lubricant film between the sliding surfaces which considerably lowers the coefficient of friction and wear loss [38,84]. Thus, GNPs because of their load bearing capability in metal matrices and ability to form a stable lubricating tribofilm render reduced shear forces, resulting in a lesser degree of plastic deformation beneath the surface mating area, hence improving wear resistance [85]. However, this valuable contribution would be decreased if GNPs agglomeration and aggregation occur [86]. Thus, 0.25 wt% GNP-Ti composites produced via dry ball milling have displayed good tribological performance for their safe and effective use in applications demanding wear and friction control. 

## 4. Conclusions

In the present research, 0.25 wt% GNP-Ti composites prepared via powder metallurgy route were investigated regarding the influence of composite constituents mixing methods upon composite properties. Dry ball milling, wet ball milling, and rotator mixing approaches were employed, and their link with morphological evolution of composite powder, sinterability, mechanical strength, and tribological performance were explored. Major outcomes are summarized below:

- The morphological study shows better homogenization of composite constituents for dry ball milled composite powders owing to the adherence of GNPs to Ti particles as a result of impact forces. GNPs aggregation for wet ball milled and rotator mixed has been observed.
- Density and sinterability of composites produced through the dry ball milling method display higher values due to the better sintering kinetics resulting from the particles’ flattened morphology, good combination of small and large particles, and well dispersed GNPs.
- Composites processed via dry ball milling show better mechanical strength, as the micro Vickers hardness is 4.56% and 15.7% higher than that of wet ball milled and rotator mixed composites, respectively. This improvement is due to the relatively denser composite and the greater role of the strengthening mechanisms as a result of GNPs’ uniform dispersion.
- The self-lubricating characteristics of GNPs have a key role in improving the tribological properties of 0.25 wt% GNP-Ti composites through the protective tribofilm formation. The effect is more pronounced in the case of dry ball milled composites due to well homogenized GNPs in the Ti matrix.
- For effective use in aerospace and tribological applications, 0.25GNP-Ti composites with improved mechanical and tribological performance have been synthesized via the powder metallurgy route by adopting the homogenizing approach of dry ball milling.

## Figures and Tables

**Table 1:** Chemical composition of as received Titanium powder.

Element Concentration (%)		
Fe	Al	Ti
0.036	0.021	99.943

**Table 2:** Processing parameters for 0.25 wt% GNP-Ti composites.

Sample Batch	Composite Constituents Mixing					Consolidation				
	Method	Medium	Charge Ratio	Speed	Time	Compaction Pressure	Sintering Temperature	Sintering Time	Heating and Cooling Rate	Sintering Environment
1	Dry ball milling	Tungsten carbide balls	Balls to powder ratio 6:1	225 rpm	3 h	400 MPa	1100 °C	2 h	10 °C/min	10^−3^ vacuum
2	Wet ball milling	Tungsten carbide balls	Balls to powder ratio 6:1	225 rpm	3 h					
3	Rotator mixing	Stainless steel blade	Volume filled 1/3	300 rpm	3 h					

**Table 3:** Density and sinterability of 0.25 wt% GNP-Ti composites.

Composites Processed via	Theoretical Density (g/cm^3^)	Green Density		Sintered Density		Sinterability
		Actual (g/cm^3^)	Relative (%)	Actual (g/cm^3^)	Relative (%)	
Dry ball milling	4.53	3.8 ± 0.1	84.0 ± 2.0	4.4 ± 0.1	97.0 ± 2.0	0.82 ± 0.04
Wet ball milling	4.53	3.5 ± 0.1	77.0 ± 2.0	4.2 ± 0.1	93.0 ± 2.0	0.68 ± 0.04
Rotator mixing	4.53	3.5 ± 0.1	77.0 ± 2.0	4.3 ± 0.1	95.0 ± 2.0	0.78 ± 0.04

**Table 4:** Density and porosity in sintered GNP-Ti composites.

Composite System	Processing	Relative Density (RD) (%)	Porosity (%) (100-%RD)	References
0.25 wt% GNP-Ti	Dry ball milling	97.0 ± 2.0	3.0 ± 2.0	This study
	Cold compaction			
	Vacuum sintering			
0.25 wt% GNP-Ti	Wet ball milling	93.0 ± 2.0	7.0 ± 2.0	This study
	Cold compaction			
	Vacuum sintering			
0.25 wt% GNP-Ti	Rotator mixing	95.0 ± 2.0	5 ± 2.0	This study
	Cold compaction			
	Vacuum sintering			
0.30 wt% GNP-Ti	Dry ball milling	96.67	3.33	[33]
	Cold compaction			
	Vacuum sintering			
0.30 wt% GNP-Ti	Dry ball milling	95	5	[30]
	Cold compaction			
	Microwave sintering			

**Table 5:** Hardness of GNP-Ti composites.

Composite System	Processing	Hardness	References
0.25 wt% GNP-Ti	Dry ball milling	440 ± 20 HV_0.1_	This study
	Cold compaction		
	Vacuum sintering		
0.25 wt% GNP-Ti	Wet ball milling	420 ± 20 HV_0.1_	This study
	Cold compaction		
	Vacuum sintering		
0.25 wt% GNP-Ti	Rotator mixing	380 ± 20 HV_0.1_	This study
	Cold compaction		
	Vacuum sintering		
0.30 wt% GNP-Ti	Dry ball milling	519 ± 35 HV_0.5_	[33]
	Cold compaction		
	Vacuum sintering		
0.30 wt% GNP-Ti	Dry ball milling	435 ± 28 HV_1_	[57]
	Spark plasma sintering		

**Table 6:** Tribological results obtained by the wear test performed through pin-on-disk tribometer.

Composites Produced through	Wear Loss (g)	Wear Rate mm^3^/Nm	Coefficient of Friction
Dry ball milling	0.0013 ± 0.0002	0.00012	0.350
Wet ball milling	0.0072 ± 0.0002	0.00084	0.413
Rotator mixing	0.0063 ± 0.002	0.00072	0.415

## Data Availability

The data is presented in this research article.
